# Higher levels of neurofilament light chain and total tau in CSF are associated with negative outcome after shunt surgery in patients with normal pressure hydrocephalus

**DOI:** 10.1186/s12987-022-00306-2

**Published:** 2022-02-14

**Authors:** Madelene Braun, Caroline Bjurnemark, Woosung Seo, Eva Freyhult, Dag Nyholm, Valter Niemelä, Kaj Blennow, Henrik Zetterberg, David Fällmar, Kim Kultima, Johan Virhammar

**Affiliations:** 1 Department of Neuroscience, Neurology, Uppsala University, Akademiska sjukhuset, ing 85, 751 85 Uppsala, Sweden; 2 Department of Medical Sciences, Clinical Chemistry, Uppsala University, Uppsala, Sweden; 3 Department of Surgical Sciences, Radiology, Uppsala University, Uppsala, Sweden; 4 Department of Cell and Molecular Biology, Uppsala University, Uppsala, Sweden; 5 Department of Psychiatry and Neurochemistry, Institute of Neuroscience and Physiology, the Sahlgrenska Academy at the University of Gothenburg, Mölndal, Sweden; 6 Clinical Neurochemistry Laboratory, Sahlgrenska University Hospital, Mölndal, Sweden; 7 Department of Neurodegenerative Disease, UCL Institute of Neurology, Queen Square, London, UK; 8 UK Dementia Research Institute at UCL, London, UK; 9 Hong Kong Center for Neurodegenerative Diseases, Hong Kong, China

**Keywords:** Normal pressure hydrocephalus, Tau, Neurofilament light chain, Amyloid beta, Biomarkers, Cerebrospinal fluid, Lumbar puncture

## Abstract

**Background:**

Lumbar punctures are a common examination in the work-up of patients with idiopathic normal pressure hydrocephalus (iNPH) and cerebrospinal fluid (CSF) biomarkers should therefore be available for use in selection of shunt candidates. The aim of this study was to investigate if CSF biomarkers are associated with outcome after shunt surgery alone or in combination with comorbidity and imaging markers, and investigate associations between CSF biomarkers and symptoms.

**Methods:**

Preoperative CSF biomarkers were analyzed in 455 patients operated with shunt surgery for iNPH at a single center during 2011–2018. Symptoms before and 12 months after shunt surgery were graded with the Swedish iNPH scale. Neurofilament light chain protein (NfL), total tau (T-tau), phosphorylated tau (P-tau) and amyloid beta1-42 (Aβ1-42) CSF levels were measured. Evans’ index and disproportionately enlarged subarachnoid space hydrocephalus were measured on preoperative CT-scans. Preoperative evaluation and follow-up 12 months after shunt surgery were available in 376 patients.

**Results:**

Higher levels of NfL and T-tau were associated with less improvement after shunt surgery (β = − 3.10, p = 0.016 and β = − 2.45, p = 0.012, respectively). Patients whose symptoms deteriorated after shunt surgery had higher preoperative levels of NfL (1250 ng/L [IQR:1020–2220] vs. 1020 [770–1649], p < 0.001) and T-tau (221 ng/L [IQR: 159–346] vs. 190 [135–261], p = 0.0039) than patients with postoperative improvement on the iNPH scale. Among the patients who improved ≥ 5 levels on the iNPH scale (55%), NfL was abnormal in 22%, T-tau in 14%, P-tau in 6% and Aβ1-42 in 45%, compared with normal reference limits. The inclusion of CSF biomarkers, imaging markers and comorbidity in multivariate predictive Orthogonal Projections to Latent Structures (OPLS) models to did not improve predictability in outcome after shunt surgery.

**Conclusions:**

Higher levels of T-tau and NfL were associated with a less favorable response to shunt surgery, suggesting a more active neurodegeneration in this group of patients. However, CSF levels of these biomarkers can be elevated also in patients who respond to shunt surgery. Thus, none of these CSF biomarkers, alone or used in combination, are suitable for excluding patients from surgery.

**Supplementary Information:**

The online version contains supplementary material available at 10.1186/s12987-022-00306-2.

## Background

Idiopathic normal pressure hydrocephalus (iNPH) is characterized by a progressing gait disturbance, cognitive impairment and urgency incontinence. The condition is most often diagnosed in patients older than 60 years and prevalence increases with age [1, 2]. Implantation of a shunt system reduces symptoms in 60–80% of patients [3–5]. Various examinations, such as magnetic resonance imaging of the brain and lumbar infusion tests, are used to aid the selection of patients for shunt surgery. Tests that reliably predict a negative outcome after shunt surgery are lacking and there are no cerebrospinal fluid (CSF) biomarkers that can identify patients who would or would not benefit from shunt insertion [6].

CSF neurofilament light chain (NfL) is a marker of degeneration of myelinated axons and is increased in neurodegenerative, neuroinflammatory, traumatic and cerebrovascular disorders [7]. Greater impairment is associated with higher levels of NfL in both patients with iNPH and patients with secondary NPH [8] and a postoperative reduction in NfL correlates with clinical improvement after shunt surgery [8].

Studies investigating the predictive value of NfL, total tau (T-tau) and amyloid beta1-42 (Aβ1-42) in lumbar CSF have most commonly been small, single-center studies including a limited number of patients [9, 10].

It has been suggested that analysis of T-tau and Aβ1-42 can differentiate between Alzheimer’s disease (AD) and iNPH and could therefore have a diagnostic value in the work-up of iNPH [11–14]. The predictive value of these biomarkers for shunt responsiveness has been tested in previous studies with diverging results [10, 15–17]. Tau is thought to reflect neuroaxonal degeneration and it has been suggested that patients with high T-tau and phosphorylated tau (P-tau) and/or low Aβ1-42 (reflecting sequestration of the protein in the brain in amyloid plaques) would be poor surgical candidates, but there is a large variation in the literature describing outcomes in this group [17, 18].

Lumbar punctures are a common examination in the work-up of patients with iNPH and routine at many centers. Analyses of CSF can thus easily be made in this group of patients. The clinical value of CSF biomarkers in patients with iNPH is not clear, with diverging results in previous studies, which have often had small sample sizes. In the present study, we aimed to investigate the value of NfL, T-tau, P-tau and Aβ1-42 to predict outcome after shunt surgery and the association between the biomarkers and clinical symptoms.

## Methods

### Study design and patients

This was a retrospective single-center study including patients with iNPH who were treated with implantation of a shunt system during January 2011 to August 2018. CSF samples were collected preoperatively and evaluations of clinical symptoms were performed at the time of CSF sampling and 3 and 12 months after shunt surgery. Outcome after surgery in the primary statistical analyses was decided at 12 months after shunt surgery but results from 3 months after shunt surgery are included in the Supplementary results. Median time between CSF sampling and shunt surgery was 6 months [IQR 4–8] and between shunt surgery and 12 months follow-up it was 12 months [IQR 12–13].

### Inclusion and exclusion criteria

**Fig. 1:**
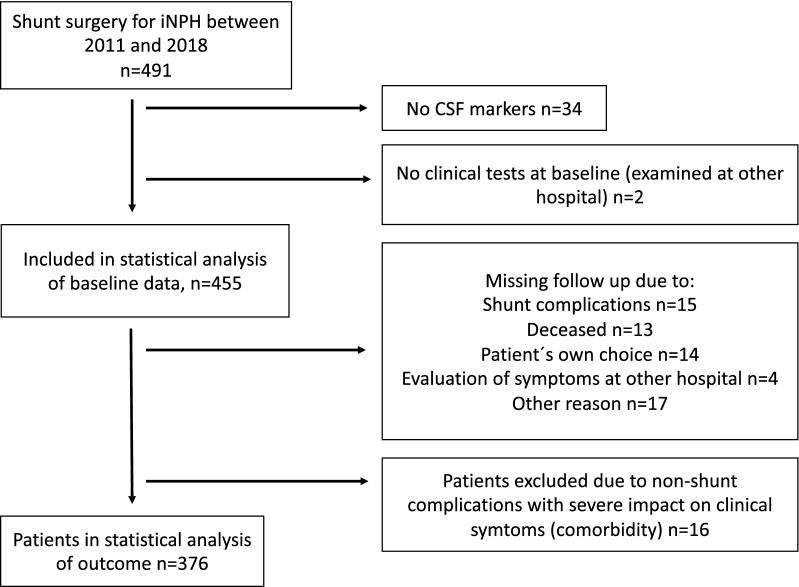
Flowchart of patients included and excluded. A total of 376 patients had pre- and postoperative evaluations and were included in the statistical analysis of outcome

Included patients met the diagnostic criteria of iNPH according to international guidelines [19]. Inclusion criteria were at least one analyzed CSF marker (NfL, T-tau, P-tau or Aß1-42) preoperatively and evaluation of symptoms before surgery with at least one domain from the iNPH scale or cognitive assessment with Mini Mental State Examination (MMSE). For analyses that included outcome after shunting, only patients with documented evaluation of symptoms preoperatively and at follow-up 12 months after shunt surgery (with at least one domain from the iNPH scale) were included. Other reasons for exclusion from analyses of outcome were follow-up at other hospitals, death before follow-up or any concomitant disease that made evaluations of outcome difficult, Fig. 1.

### Clinical evaluations

All patients were examined by a specialized team including neurologists, neurosurgeons, nurses, physiotherapists and occupational therapists. The work-up included CSF tap tests and lumbar infusion tests. Symptoms were graded using the MMSE and the iNPH scale [20]. The iNPH scale is based on grading of four major symptom domains (gait, balance, cognitive function and continence) and is used to assess a patient’s disability. Each domain is scored between 0 and 100, where 0 represents the most severe state possible and 100 is the performance of an age-matched healthy individual. A total score is calculated as the average of the four domains (gait has double weight), or as the average of the available domains if any domain is missing. A subgroup of 162 (36%) patients was examined with an alternative version of the Stroop color test and Stroop interference including only 24 colored circles/words instead of 100 squares as in the original iNPH scale. For these patients, the cognitive domain score was calculated using a modified conversion table (Additional file 1: Table S1). Outcome after shunting was calculated as the difference in iNPH scale score between 12 months follow-up post-surgery and the preoperative visit, referred to as delta iNPH scale. Outcome at 3 months after shunt surgery was included in the supplement.

Information regarding the following comorbidities was documented: history of ischemic stroke or transient ischemic attack, coronary heart disease defined as previous acute coronary syndrome, diabetes mellitus, oral anticoagulants and hypertension. Hypertension was recorded if the patient was on medication for hypertension, Table 1.

**Table 1:** Demographical data and preoperative symptoms

	n	
Age, years (range)	455	75 (50–89)
Sex, male (%)	455	257 (57)
Time from examination to shunt, months, median (IQR)	455	6 (4–8)
Time from operation to follow-up, months, median (IQR)	376	12 (12–13)
*Comorbidities*		
Diabetes, n (%)	455	115 (25)
Hypertension, n (%)	455	291 (64)
Hyperlipidemia, n (%)	455	160 (35)
Ischemic stroke or transient ischemic attack, n (%)	455	53 (12)
Cardiovascular disease, n (%)	455	57 (13)
Oral anticoagulants, n (%)	455	35 (8)
*Preoperative symptoms*		
Total iNPH scale, median (IQR)	453	50 (37–62)
Motor domain, median (IQR)	451	32 (18–55)
Cognitive domain, median (IQR)	265	50 (33–67)
Continence domain, median (IQR)	439	60 (40–80)
Balance domain, median (IQR)	442	67 (67–83)
MMSE, median (IQR)	446	25 (21–27)
Evans’ index, median (IQR)	455	0.37 (0.34–0.40)
DESH, n (%)	455	382 (84)

*IQR* interquartile range, *iNPH* idiopathic normal pressure hydrocephalus; *MMSE* mini-mental state examination, *DESH* disproportionately enlarged subarachnoid-space hydrocephalus

### Laboratory analyses

Levels of NfL, T-tau, P-tau and Aβ1-42 were analyzed from preoperative CSF samples. Samples were collected into polypropylene tubes and the first 3 mL of the lumbar tap were used for analyses. Samples were analyzed at the Clinical Neurochemistry Laboratory at Sahlgrenska University Hospital and laboratory technicians were blinded to clinical data. NfL level was measured using a commercially available enzyme-linked immunosorbent assay, in accordance with instructions from the manufacturer (UmanDiagnostics, Umeå, Sweden). T-tau, P-tau and Aβ1-42 levels were measured using INNOTEST enzyme-linked immunosorbent assays in accordance with instructions from the manufacturer (Fujirebio, Ghent, Belgium). Procedures for acquiring high longitudinal stability in the measurements has been described previously and the coefficients of variation for the quality-control samples from the same lab was in a previous study between 7.1 and 11.5% for T-tau, P-tau, and Aβ1-42 [21].

Laboratory-specific normal reference ranges: NfL: 40–60 years, < 890 ng/L, > 60 years, < 1850 ng/L [22, 23]; T-tau: < 350 ng/L; P-tau: < 60 ng/L; Aβ1-42: > 530 ng/L [23].

### Imaging markers

Evans’ index and disproportionately enlarged subarachnoid space hydrocephalus (DESH) was assessed on preoperative computed tomography (CT) scans of the brain (CT in 430 patients and MRI in 25 patients). The scans were performed median 1 day (IQR: 1–3) before the shunt surgery. Evans’ index and DESH were assessed as previously described [24, 25]. Evans’ index was included as a covariate in all regression analyses as a measure of ventricular volume while DESH was analyzed as a separate predictive variable.

### Statistical analysis

The biomarker values were log_2_-transformed before all analyses to not violate assumption of normality. Also, time to shunt (in months) was log2-transformed after adding a pseudo-count of 1. To investigate the association between surgery outcome (delta iNPH scale) and biomarker levels, a linear regression analysis was performed, adjusting for age, sex, waiting time for shunt surgery, iNPH-scale score at baseline and Evans’ index. Each CSF biomarker and DESH was analyzed separately, with shunt surgery outcome as the dependent variable. The regression coefficient β for the biomarker was calculated and assessed using a t-test. As the biomarker level is on the log_2_ scale, the beta value is the expected increase in delta iNPH scale when the biomarker level is doubled. Multivariate predictive orthogonal projections to latent structures (OPLS) models [26] predicting outcome 12 months after surgery were constructed: (1) based on sets of basic clinical data (base) including: age, sex, waiting time for shunt surgery, iNPH score at baseline and presence of selected comorbidities (diabetes mellitus, hyperlipidemia, hypertension and previous stroke/TIA or myocardial infarction,); (2) basic data with the addition of the imaging markers Evans’ index and DESH; (3) basic data with addition of levels of markers in CSF (NfL, T-tau, P-tau and Aβ1-42), and (4) finally all above listed variables together, using the R-package ropls [27].

To investigate associations between levels of biomarkers and preoperative symptoms at baseline, linear models were fitted, adjusting for age, sex and Evans’ index. The significance was assessed using a t-test. Differences in levels of biomarkers between patients with deterioration and patients with improvement were analyzed with the Mann Whitney U-test. Reliability between the two versions of the Stroop tests was tested with an intraclass correlation coefficient. Analyses were performed using R version 4.0.2 and SPSS version 27; p < 0.05 was considered significant.

## Results

During the study period, 491 patients (257 male [57%]) with iNPH were operated with shunt implantation. Levels of at least one CSF marker and evaluation of symptoms at baseline were available in 455 patients, who were included in the statistical analysis. Median age at time of shunt surgery was 75 years (range 50–89). Two hundred forty-five of 376 patients (65%) improved > 0 points on the iNPH scale in analyses of outcome and 205 (55%) improved ≥ 5 points (delta iNPH scale > 5). The median preoperative iNPH scale score was 50 [IQR: 37–62] and the median score 12 months after shunt surgery 56 [IQR: 41–73]. Symptoms and comorbidities are presented in Table 1. The preoperative iNPH scale score was lower (more severe symptoms) in the 79 patients excluded from analyses of outcome compared with the included 376 included patients (44 (IQR: 31–58) vs 50 (IQR: 38–63), p = 0.016). There was no difference between included and excluded patients regarding other descriptive data.

NfL levels were higher than the reference range in 67 (29%) of all patients and in 25 (22%) of shunt responders (delta iNPH > 5 points). The proportions of patients with levels of CSF biomarkers outside reference ranges are presented in Table 2. There was a significant negative association between shunt surgery outcome 12 months post-operative (delta iNPH scale) with higher levels of NfL (β = -3.10, p = 0.016) and T-tau (β = -2.45, p = 0.012). There were no associations between levels of P-tau or Aβ1-42 with outcome after shunt surgery 12 months post operatively. There was a trend, but no significant association, between DESH and outcome after shunt surgery (β = 4.30, p = 0.055), Table 3. The models were adjusted for age, sex, waiting time for shunt surgery, preoperative iNPH scale score and Evans’ index, Table 3. Associations between CSF biomarkers and outcome 3 months after shunt surgery are presented in Additional file 1: Table S2. At 3 months after shunting there was a significant association between shunt surgery outcome with levels of NfL (β = − 2.22, p = 0.026) and Aβ1-42 (β = − 3.95, p = 0.0013). Only 187 patients in the analyses of outcome were investigated with preoperative NfL compared with 364 who were investigated with T-tau. Therefore, the association between CSF biomarkers and outcome after shunt surgery (12 months) for all CSF markers and DESH were also investigated only in the individuals with NfL data. In this analysis the association between T-tau and Aβ1-42 with outcome were slightly weaker, β = − 1.66 (p = 0.22) and β = 3.14 (p = 0.12), respectively, Additional file 1: Table S3.

**Table 2:** Median CSF biomarker levels in all patients and proportion of patients with levels outside reference range in the whole sample and in patients with postoperative improvement

	n	Median (IQR)	Outside reference range, n (%)	Outside reference range among shunt responders^1^, n (%)
NfL, ng/L	234	1175 (850–1970)	67 (29)	25 (22)
T-tau, ng/L	442	200 (145–282)	73 (17)	29 (14)
P-tau, ng/L	436	29 (23–37)	30 (7)	12 (6)
Aβ1–42, ng/L	438	536 (386–695)	215 (49)	93 (45)

*CSF* cerebrospinal fluid, *NfL* neurofilament light protein, *T-tau* total tau, *P-tau* phosphorylated tau, *Aβ1–42* amyloid beta1-42, *IQR* interquartile range

Normal reference ranges: Aβ1–42: > 530 ng/L; NfL: 40–60 years, < 890 ng/L, > 60 years, < 1850 ng/L; T-tau: < 350 ng/L; P-Tau: < 60 ng/L

^1^Improvement ≥ 5 points on iNPH scale 12 months after shunt surgery

**Table 3:** A linear model was built for each predictor relative to the outcome 12 months postoperative change on the iNPH scale (delta iNPH scale) at follow-up and the covariates age, sex, waiting time for shunt surgery, iNPH scale score at baseline and Evans’ index

	Coefficient	Analysis of variance P-value
NfL (n = 187)	− 3.10	**0.016**
T-tau (n = 364)	− 2.45	**0.012**
P-tau (n = 362)	− 0.62	0.65
Aβ1–42 (n = 363)	2.76	0.058
DESH (n = 376)	4.30	0.055
Covariates:		
Age	− 0.50	** < 0.001**
Sex	− 1.39	0.42
Time to shunt	− 6.03	** < 0.001**
iNPH scale baseline	− 0.23	** < 0.001**
Evans´ index	20.08	0.33

*iNPH* idiopathic normal pressure hydrocephalus, *NfL* neurofilament light protein, *T-tau* total tau, *P-tau* phosphorylated tau, *Aβ1–42* amyloid beta-42; *DESH* disproportionately enlarged subarachnoid-space hydrocephalus

Bold numbers are significant

**Fig. 2:**
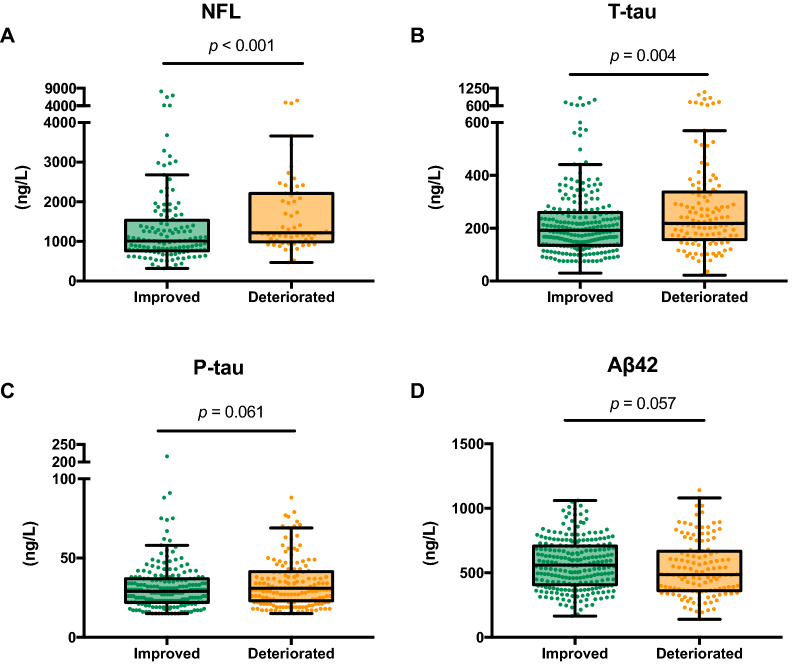
Levels of cerebrospinal fluid biomarkers in patients with improved postoperative idiopathic normal pressure hydrocephalus scale (delta iNPH scale > 0) at follow-up and patients who deteriorated (delta iNPH scale < 0). Differences analyzed with the Mann Whitney U-test

NfL was higher in patients who deteriorated after shunt surgery 12 months postoperative than in patients with postoperative iNPH scale improvement (1250 [IQR:1020–2220] vs. 1020 [770–1649], p < 0.001). T-tau was higher in patients who deteriorated after shunt surgery than in patients with postoperative iNPH scale improvement (221 [IQR: 159–346] vs. 190 [135–261], p = 0.0039). There was no significant difference in P-tau or Aβ1-42 between patients who deteriorated and patients who did not, p = 0.061 and p = 0.057, respectively, Fig. 2.

**Fig. 3:**
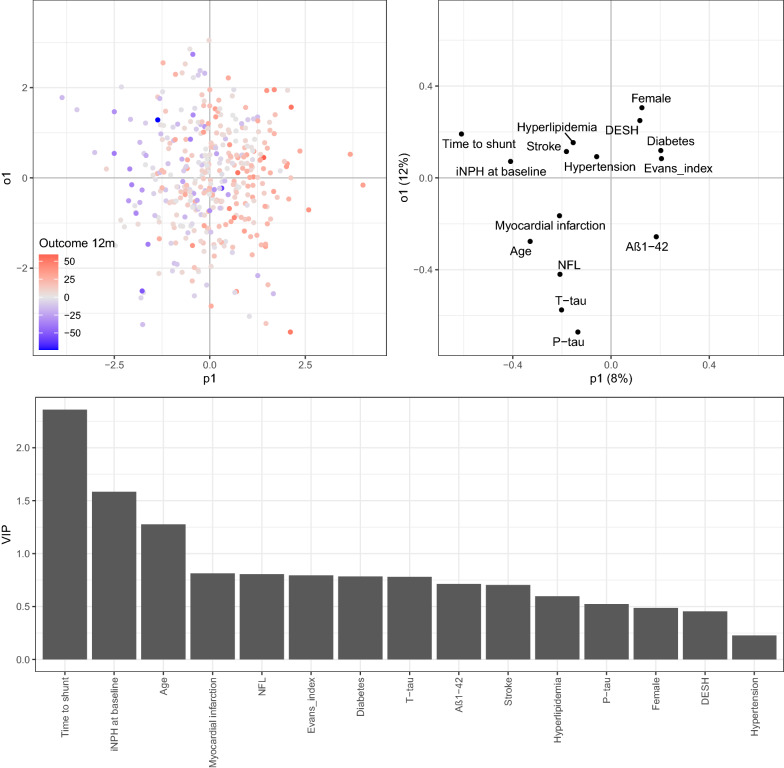
OPLS model with both base variables, comorbidity, imaging markers and CSF biomarkers included, built on prediction of outcome 12 months after shunt surgery (delta iNPH scale). The upper left image shows the score plot that illustrate the sample separation, predictive component along the x-axis and orthogonal component along y-axis. The samples are colored according to their delta iNPH value at 12 months. The upper right image shows the loading plot, with the predictive component shown along the x-axis. Variables to the left (with decreasing values) are more associated with negative outcome while variables to the right are associated with positive outcome after shunt surgery. In the bottom, VIP-values illustrating the predictive effect of each variable included in the model. iNPH at baseline refers to preoperative iNPH scale score; *DESH* disproportionately enlarged subarachnoid-space hydrocephalus, *NfL* neurofilament light protein, *T-tau* total tau, *P-tau* phosphorylated tau, *Aβ1–42* amyloid beta1–42

To investigate if basic clinical data including comorbidities with the addition of imaging markers and CSF markers can be used to improve surgery outcome prediction 12 months after surgery, four OPLS models were trained, stepwise adding variables to the models. At follow-up 12 months after shunt surgery the base model (base data + comorbidities) showed a weak association to surgery outcome, as can be seen in the cross validated score (pQ2 < 0.01, Q2 = 0.156). The most important variable was waiting time for shunt surgery (VIP = 2.08), Table 4 and Additional file 1: Fig. S1. Adding additional variables to the model did not improve performance in predicting surgery outcome, as can be seen by no major improvements in pQ2 was achieved. A very weak improvement was found adding imaging markers (pQ2 < 0.01, Q = 0. 16) compared with only base data (Additional file 1: Fig. S2), but adding CSF markers did not improve the model (pQ2 < 0.01, Q = 0.151), Additional file 1: Fig. S3. In all models, age, waiting time to shunt surgery and iNPH score at baseline were the most important variables associated with outcome after shunt surgery (highest VIP). Figure 3 illustrates the model with all variables included.

**Table 4:** Four OPLS predicting outcome 12 months after shunt surgery

Variables:	R2Y	Q2	pR2Y	pQ2
1. Base	0.192	0.156	0.01	0.01
2. Base + Evans’ index + DESH	0.203	0.16	0.01	0.01
3. Base + CSF markers	0.22	0.151	0.01	0.01
4. Base + Evans + DESH + CSF markers	0.231	0.157	0.01	0.01

The first model (1) based on basic clinical data (base) including: age, sex, waiting time for shunt surgery, iNPH score at baseline and presence the comorbidities: diabetes mellitus, hyperlipidemia, hypertension and previous stroke or myocardial infarction; (2) basic data with the addition of the imaging markers Evans’ index and DESH; (3) basic data with addition of levels of markers in CSF (NfL, T-tau, P-tau and Aβ1-42), and (4) finally all above listed variables together

*iNPH* idiopathic normal pressure hydrocephalus, *NfL* neurofilament light protein, *T-tau* total tau, *P-tau* phosphorylated tau, *Aβ1–42* amyloid beta-42, *DESH* disproportionately enlarged subarachnoid-space hydrocephalus

There were associations between higher NfL and lower total score on the iNPH scale at baseline. There was an association between higher Aβ1-42 and better cognitive function at baseline, whether this was measured with MMSE or the cognitive domain of the iNPH scale. All associations between symptoms at baseline and CSF markers are presented in Table 5.

**Table 5:** Coefficients from a linear regression model for each CSF marker with preoperative symptoms at baseline as outcome and the covariates age, sex and Evan´s index

CSF marker	Total iNPH scale	Motor domain	Cognitive domain	Balance domain	Continence domain	MMSE
NfL	− 4.24 ***	− 5.23***	-1.80	− 3.73*	− 4.65**	− 0.66
T-tau	− 0.24	0.50	1.63	− 0.15	− 1.54	− 0.32
P-tau	3.26*	4.57*	3.42	− 0.78	4.31*	− 0.22
Aβ1–42	0.86	− 0.32	8.24***	1.42	0.73	1.62***

*CSF* cerebrospinal fluid, *iNPH* idiopathic normal pressure hydrocephalus, *NfL* neurofilament light protein, *T-tau* total tau, *P-tau* phosphorylated tau, *Aβ1–42* amyloid beta1–42

Bold numbers are significant. * < 0.05; ** < 0.01; *** < 0.001

The reliability, tested with intraclass correlation coefficient between the original Stroop tests and the alternative versions with only 24 colored circles/words, was 0.89 for Stroop color and 0.73 for Stroop interference.

## Discussion

This is, to our knowledge, the largest study assessing the association between preoperative CSF biomarkers with outcome after shunt surgery in patients with iNPH. In this single-center study of 455 patients with iNPH, of whom 376 were examined before and 12 months after shunt surgery, levels of CSF biomarkers were compared with outcome after shunt surgery and symptoms at baseline. Higher preoperative levels of NfL and T-tau in CSF were associated with less improvement one year after shunt surgery. Patients who deteriorated after surgery had higher preoperative levels of NfL and T-tau. However, half of the patients who improved after shunt surgery had preoperative levels of Aβ1-42 below reference ranges, and NfL was above reference range in 22% of the shunt responders. Combining CSF biomarkers with imaging data and data of comorbidity did not improve the ability to predict outcome after shunt surgery. This indicates that the CSF markers included here are not suitable for excluding patients from shunt surgery.

### Predictive value of CSF biomarkers

This study indicated an association between higher preoperative levels of NfL and poorer outcome measured with the iNPH scale. This is in line with a study by Tullberg et al. reporting that NfL may be a clinically useful biomarker for good prognosis in iNPH [28].

High NfL levels have previously been described in patients with NPH, but also frontotemporal dementia, AD and vascular dementia [29]. The association between high NfL and worse outcome in iNPH could therefore be explained by comorbidity with other neurodegenerative disorders in some patients. Cerebrovascular disease is common in iNPH and it is possible that concomitant vascular disease is the explanation for both increased NfL and its association with less improvement after shunt surgery [29, 30].

There was also an association between higher preoperative levels of T-tau and a less favorable outcome one year after shunt. There was no association between P-tau and outcome after shunting. This is consistent with most previous studies, which have reported no difference in P-Tau between patients who deteriorated and patients who did not [12]. However, one study reported that levels of P-tau were associated with cognitive outcome after shunting [31] In line with our results, increased T-tau has been suggested to predict less postoperative improvement in gait function [18]. The levels of T-tau and P-tau have previously been compared between AD and iNPH, and found to be higher in AD than in iNPH [18]. Furthermore, T-tau is lower in iNPH than in healthy elderly subjects, at a group level [32]. It therefore seems possible that individuals with increased T-tau and P-tau with iNPH might suffer from neurodegenerative comorbidity. This could be explained by the tau-pathogenesis hypothesis [33]. T-tau and P-tau are microtubule-associated proteins found in the cytoskeleton of mainly non-myelinated axons in the CNS. This structure is seen in cortical areas of the brain, especially in regions involved in memory function, such as the limbic cortex, including the hippocampus [33, 34].

The association between Aβ1-42 and shunt surgery outcome was not as strong as those for NfL and T-tau. A previous study reported that Aβ1-42 could predict outcome at 6-month follow-up and that a combination of Aβ1-42 and T-tau could predict outcome after shunting. Differences compared with our study were a relatively small sample size and that their samples were collected from the ventricles [17]. Levels of Aβ1-42 are decreased in AD, but in previous studies have also been shown to be lower in iNPH than in healthy controls [18, 32]. It has therefore been questioned whether Aβ1-42 could aid discrimination between iNPH and AD [15, 18, 32]. One study also links low levels of Aβ1-42 to a more definitive cognitive impairment that could suggest AD pathology [35]. In AD, there is a selective reduction of Aβ1-42 compared with the shorter and more hydrophilic Aβ1-38 and Aβ1-40 peptides in CSF [36]. This is due to sequestration of Aβ1-42 in amyloid plaques in the brain. In iNPH, on the other hand, there is a concordant reduction in Aβ1-38, Aβ1-40 and Aβ1-42 [36]. The molecular mechanism is unknown, but the levels normalize following successful shunt surgery [36]. Hence, CSF Aβ1-42/Aβ1-40 is a better marker for Aβ plaque pathology in conditions in which disturbed CSF dynamics can be expected. Unfortunately, we did not have access to CSF Aβ1-38 or Aβ1-40 measures in the current study.

### Associations between CSF biomarkers and preoperative symptoms

Higher NfL was associated with lower scores on the total iNPH scale as well as on the separate motor, balance and continence domains (Table 5). Possible explanations include white matter injuries from compressing enlarged ventricles, or trans-ependymal CSF passage with excessive white matter edema or concomitant cerebrovascular disease with white matter lesions [28, 30]. In line with our results, previous studies have found that preoperative NfL levels correlate with the severity of clinical symptoms [8, 12, 28]. We found an association, after controlling for age and sex, between lower Aβ1-42 and cognitive impairment, measured with either MMSE or the cognitive domain from the iNPH scale. This could be related to comorbidity with neurodegenerative disease such as AD, as mentioned above, and is in congruence with previous studies of both CSF and cortical biopsies [37–39].

### Outcome in patients with pathological levels of CSF markers

Although there were associations between a less favorable outcome and high levels of NfL and T-tau, a majority of the patients with pathologically altered levels of the CSF biomarkers responded well to shunt surgery. To investigate if basic clinical data, including comorbidities, imaging markers and CSF markers can be used to improve surgery outcome prediction we stepwise trained a set of OPLS models. In all models, age, waiting time to shunt surgery and iNPH score at baseline were the most important variables associated with outcome after shunt surgery. This strengthens our suggestion that the now investigated CSF biomarkers may have differential diagnostic value, but patients should not be excluded from shunt surgery because of abnormal CSF biomarker levels.

Some limitations should be considered. There were long waiting times for shunt surgery at our center during the study period, due to insufficient capacity. Long waiting times negatively affect the outcome after shunt surgery [40, 41] and patients deteriorate while waiting for surgery. No new clinical assessments were performed right before shunt surgery. This is the probable cause for the lower proportion of patients who improved in this study (55%) compared with in previous literature. This also explains why waiting time for surgery was the variable with strongest association with outcome. Furthermore, outcome after shunting is most favorable when the time from onset of symptoms to surgery is minimized [42]. In the present material, time from onset of symptoms to examination was estimated to be 1–8 years but was not included in the statistical analysis, since this variable can be uncertain and requires reliable information from a relative. The follow-up at 12 months after shunt surgery is late compared with in many studies in the field, but an even longer follow-up time might have revealed more concomitant neurodegenerative disorders. Unfortunately, we did not have data that extended longer than 12 months. However, the purpose of this study was to investigate associations between levels of biomarkers with outcome of shunt surgery and we believe that 12 months follow-up is sufficient to assess the result of shunt surgery.

We did not have data of years of education, that may be useful data when interpreting results of cognitive testing. In a retrospective study such as this one, there is always a risk of inclusion bias since some patients may have been excluded from shunt surgery (and therefore this study) based on highly pathological levels of the CSF biomarkers. Furthermore, patients excluded from the outcome analyses had more severe preoperative symptoms.

Two radiological measures were included. However, white matter changes that are believed to represent cerebrovascular changes have been associated with deterioration after surgery [43]. The association between high NfL and less favorable outcome could also be attributed to more pronounced white matter brain lesions.

## Conclusions

Higher levels of NfL and T-tau are associated with a less favorable response to shunt surgery. High levels of NfL were associated with more pronounced motor symptoms before surgery and lower levels of Aβ1-42 were associated with more cognitive dysfunction. These associations may be explained by concomitant subcortical vascular injuries and AD pathology, respectively. However, the majority of patients with increased CSF levels of NfL and T-tau responded to shunt surgery and inclusion of CSF biomarkers, imaging markers and comorbidity in multivariate predictive OPLS models did not improve predictability in outcome after surgery. We conclude that none of these CSF biomarkers should be used to exclude patients from surgery.

## Supplementary Information

**Additional file 1: Table S1**. Conversion table for the alternative versions of Stroop color and Stroop interference with only 24 colored circles/words. **Table S2**. A linear model was built for each predictor relative to the outcome 3 month postoperative change on the iNPH scale (delta iNPH scale 3 months) at follow-up and the covariates age, sex, waiting time for shunt surgery, iNPH scale score at baseline and Evans’ index. **Table S3**. Including only patients with preoperative NfL. A linear model was built for each predictor relative to the outcome 12-month postoperative change on the iNPH scale (delta iNPH scale) at follow-up and the covariates age, sex, waiting time for shunt surgery, iNPH scale score at baseline and Evans’ index. **Figure S1**. OPLS models built on prediction of outcome 12 months after shunt surgery (delta iNPH scale). The first model (Supplementary figure 1) based on basic clinical data (base) including: age, sex, waiting time for shunt surgery, iNPH score at baseline and presence of the comorbidities: diabetes mellitus, hyperlipidemia, hypertension and previous stroke or myocardial infarction. **Figure S2**. includes basic data with the addition of the imaging markers Evans’ index and DESH. **Figure S3**. includes basic data with addition of levels of markers in CSF (NfL, T-tau, P-tau and Aβ1-42). The model with all above variables included are included in the main manuscript (Figure 3). The upper left image shows the score plot that illustrate the sample separation, predictive component along the x-axis and orthogonal component along y-axis. The samples are colored according to their delta iNPH value at 12 months. The upper right image shows the loading plot, with the predictive component shown along the x-axis. Variables to the left (with decreasing values) are more associated with negative outcome while variables to the right are associated with positive outcome after shunt surgery. In the bottom, VIP-values illustrating the predictive effect of each variable included in the model. iNPH at baseline refers to preoperative iNPH scale score; DESH = disproportionately enlarged subarachnoid-space hydrocephalus; NfL = neurofilament light protein; T-tau = total tau; P-tau = phosphorylated tau; Aβ1-42 = amyloid beta1-42.

## Data Availability

The datasets analyzed during the current study are available from the cor- responding author on reasonable request.

## Abbreviations

iNPH: Idiopathic normal pressure hydrocephalus
CSF: Cerebrospinal fluid
NfL: Neurofilament light chain protein
T-tau: Total tau
P-tau: Phosphorylated tau
Aβ1-42: Amyloid beta1-42
IQR: Interquartile range
AD: Alzheimer’s disease
MMSE: Mini Mental State Examination
CNS: Central nervous system
DESH: Disproportionately enlarged subarachnoid space hydrocephalus
