# Supplementary material for: Higher levels of neurofilament light chain and total tau in CSF are associated with negative outcome after shunt surgery in patients with normal pressure hydrocephalus
Source: Fluids Barriers CNS. 2022 Feb 14;19:15. doi: 10.1186/s12987-022-00306-2 (PMC8845290; doi:10.1186/s12987-022-00306-2)
Supplement: Supplementary file 1 — Additional file 1: Table S1. Conversion table for the alternative versions of Stroop color and Stroop interference with only 24 colored circles/words. Table S2. A linear model was built for each predictor relative to the outcome 3 month postoperative change on the iNPH scale (delta iNPH scale 3 months) at follow-up and the covariates age, sex, waiting time for shunt surgery, iNPH scale score at baseline and Evans’ index. Table S3. Including only patients with preoperative NfL. A linear model was built for each predictor relative to the outcome 12-month postoperative change on the iNPH scale (delta iNPH scale) at follow-up and the covariates age, sex, waiting time for shunt surgery, iNPH scale score at baseline and Evans’ index. Figure S1. OPLS models built on prediction of outcome 12 months after shunt surgery (delta iNPH scale). The first model (Supplementary figure 1) based on basic clinical data (base) including: age, sex, waiting time for shunt surgery, iNPH score at baseline and presence of the comorbidities: diabetes mellitus, hyperlipidemia, hypertension and previous stroke or myocardial infarction. Figure S2. includes basic data with the addition of the imaging markers Evans’ index and DESH. Figure S3. includes basic data with addition of levels of markers in CSF (NfL, T-tau, P-tau and Aβ1-42). The model with all above variables included are included in the main manuscript (Figure 3). The upper left image shows the score plot that illustrate the sample separation, predictive component along the x-axis and orthogonal component along y-axis. The samples are colored according to their delta iNPH value at 12 months. The upper right image shows the loading plot, with the predictive component shown along the x-axis. Variables to the left (with decreasing values) are more associated with negative outcome while variables to the right are associated with positive outcome after shunt surgery. In the bottom, VIP-values illustrating the predictive effect of each variabl [file 12987_2022_306_MOESM1_ESM.docx]

Additional file 1

**Additional Methods**

In the years before the full iNPH scale was implemented at our center, a subgroup of 162 (36%) patients was examined with an alternative version of the Stroop color test and Stroop interference including only 24 colored circles/words instead of 100 colored squares as in the original iNPH scale. None of these patients were examined with the Rey Auditory Verbal Learning Test.

For these 162 patients, the domain score (0–100 points) was calculated using a modified conversion table (Additional file 1: Table S1). The conversion table was constructed using normative data from 120 patients with iNPH, and then slightly modified so that each 10-point interval, at least represented two seconds.

Twelve patients were examined with both the version with 24 circles/words and the version with 100 squares, to assess reliability between the scales. The intraclass correlation coefficient was 0.89 for Stroop color and 0.73 for Stroop interference.

**Additional file 1: Table S1**. Conversion table for the alternative versions of Stroop color and Stroop interference with only 24 colored circles/words.

| Stroop color,  24 squares |  | Stroop interference,  24 squares |  |
| --- | --- | --- | --- |
| Time (seconds) | Score | Time (seconds) | Score |
| < 12 | 100 | < 27 | 100 |
| 13–14 | 90 | 28–30 | 90 |
| 15–16 | 80 | 31–33 | 80 |
| 17–18 | 70 | 34–38 | 70 |
| 19–20 | 60 | 39–44 | 60 |
| 21–22 | 50 | 45–54 | 50 |
| 23–25 | 40 | 55–66 | 40 |
| 26–36 | 30 | 67–81 | 30 |
| 37–46 | 20 | 82–94 | 20 |
| 47–179 | 10 | > 95 | 10 |
| > 180 or fail | 0 | fail | 0 |

**Additional Results**

**Additional file 1: Table S2.** A linear model was built for each predictor relative to the outcome 3 month postoperative change on the iNPH scale (delta iNPH scale 3 months) at follow-up and the covariates age, sex, waiting time for shunt surgery, iNPH scale score at baseline and Evans’ index.

|  | Coefficient | Analysis of variance  P-value |
| --- | --- | --- |
| NfL (n=187) | -2.22 | **0.026** |
| T-tau (n=364) | -1.52 | 0.068 |
| P-tau (n=362) | -0.016 | 0.99 |
| Aβ1-42 (n=363) | 3.95 | **0.0013** |
| DESH (n=376) | 3.13 | 0.10 |
| Covariates: |  |  |
| Age | -0.44 | **< 0.001** |
| Sex | -1.60 | 0.28 |
| Time to shunt | -5.84 | **<0.001** |
| iNPH scale baseline | -0.15 | **<0.001** |
| Evans´ index | 13.49 | 0.45 |

iNPH = idiopathic normal pressure hydrocephalus; NfL = neurofilament light protein; T-tau = total tau; P-tau = phosphorylated tau; Aβ1-42 = amyloid beta-42; DESH = disproportionately enlarged subarachnoid-space hydrocephalus. Bold numbers are significant.

**Additional file 1: Table S3**. Including only patients with preoperative NfL. A linear model was built for each predictor relative to the outcome 12-month postoperative change on the iNPH scale (delta iNPH scale) at follow-up and the covariates age, sex, waiting time for shunt surgery, iNPH scale score at baseline and Evans’ index.

|  | Coefficient | Analysis of variance  P-value |
| --- | --- | --- |
| NfL (n=187) | -3.10 | **0.016** |
| T-tau (n=187) | -1.67 | 0.22 |
| P-tau (n=187) | -0.94 | 0.58 |
| Aβ1-42 (n=187) | 3.14 | 0.13 |
| DESH (n=187) | 6.44 | **0.027** |

iNPH = idiopathic normal pressure hydrocephalus; NfL = neurofilament light protein; T-tau = total tau; P-tau = phosphorylated tau; Aβ1-42 = amyloid beta-42; DESH = disproportionately enlarged subarachnoid-space hydrocephalus. Bold numbers are significant.

**Additional file 1: Figures S1–3**

OPLS models built on prediction of outcome 12 months after shunt surgery (delta iNPH scale). The first model (Additional file 1: Figure S1) based on basic clinical data (base) including: age, sex, waiting time for shunt surgery, iNPH score at baseline and presence of the comorbidities: diabetes mellitus, hyperlipidemia, hypertension and previous stroke or myocardial infarction. Additional file 1: Figure S2 includes basic data with the addition of the imaging markers Evans’ index and DESH. Additional file 1: Figure S3 includes basic data with addition of levels of markers in CSF (NfL, T-tau, P-tau and Aβ1-42). The model with all above variables included are included in the main manuscript (Figure 3). The upper left image shows the score plot that illustrate the sample separation, predictive component along the x-axis and orthogonal component along y-axis. The samples are colored according to their delta iNPH value at 12 months. The upper right image shows the loading plot, with the predictive component shown along the x-axis. Variables to the left (with decreasing values) are more associated with negative outcome while variables to the right are associated with positive outcome after shunt surgery. In the bottom, VIP-values illustrating the predictive effect of each variable included in the model. iNPH at baseline refers to preoperative iNPH scale score; DESH = disproportionately enlarged subarachnoid-space hydrocephalus; NfL = neurofilament light protein; T-tau = total tau; P-tau = phosphorylated tau; Aβ1-42 = amyloid beta1-42.

**Additional file 1: Figure S1**

**Additional file 1: Figure S2**

**Additional file 1: Figure S3**
